# Designing Gestures of Robots in Specific Fields for Different Perceived Personality Traits

**DOI:** 10.3389/fpsyg.2022.876972

**Published:** 2022-06-23

**Authors:** Jin Niu, Chih-Fu Wu, Xiao Dou, Kai-Chieh Lin

**Affiliations:** ^1^The Graduate Institute of Design Science, Tatung University, Taipei, Taiwan; ^2^Department of Industrial Design, Tatung University, Taipei, Taiwan; ^3^The College of Fine Arts, Guangdong Polytechnic Normal University, Guangzhou, China

**Keywords:** social robot, occupational field, social cue, gesture, perceived personalities

## Abstract

Since their development, social robots have been a popular topic of research, with numerous studies evaluating their functionality or task performance. In recent years, social robots have begun to be regarded as social actors at work, and their social attributes have been explored. Therefore, this study focused on four occupational fields (shopping reception, home companion, education, and security) where robots are widely used, exploring the influence of robot gestures on their perceived personality traits and comparing the gesture design guidelines required in specific occupational fields. The study was conducted in two stages. In the first stage, an interactive script was developed; moreover, observation was employed to derive gestures related to the discourse on the fields of interest. The second stage involved robot experimentation based on human–robot interaction through video. Results show that metaphoric gestures appeared less frequently than did deictic, iconic, or beat gestures. Robots’ perceived personality traits were categorized into sociality, competence, and status. Introducing all types of gestures helped enhance perceived sociality. The addition of deictic, and iconic gestures significantly improved perceived competence and perceived status. Regarding the shopping reception robot, after the inclusion of basic deictic and iconic gestures, sufficient beats gestures should be implemented to create a friendly and outgoing demeanor, thereby promoting user acceptance. In the home companion, education, and security contexts, the addition of beat gestures did not affect the overall acceptance level; the designs should instead be focused on the integration of the other gesture types.

## Introduction

In recent years, social robots have been widely applied in numerous occupations. They are not only suitable for unique, dangerous, or professional fields, but also as a part of people’s everyday lives. Social robots are used in various spaces and serve in a range of roles and tasks (e.g., in supermarkets, hospitals, schools, homes, and restaurants; Looije et al., 2010; Koceski and Koceska, 2016; Sabelli and Kanda, 2016; Benitti and Spolaôr, 2017; McGinn et al., 2017; Chang et al., 2018; Savela et al., 2018).

In human–human interaction, social perception refers to the process in which people automatically classify social groups (e.g., people of a particular gender, age group, or race) based on social cues, such as the tone of voice and gestures (Jussim, 1991). Psychologists have noted that according to role congruity theories, men are more likely to acquire leadership positions due to their assertiveness, strength, and independence, whereas women are more likely to fill communal roles due to traits of sensitivity and emotional expressiveness (Eagly et al., 2000; Ceci et al., 2009; Barth et al., 2015).

In the earliest research on human–computer interaction, some scholars reported that beyond technological products, computers possess certain social attributes. This theory was termed computers as social actor (CASA) theory (Nass et al., 1994) and is widely acknowledged by scholars in the field of human–robot interaction (HRI). Researchers have observed that when people interact with robots, they exhibit behaviors and reactions similar to those displayed when they interact with other humans (Lee et al., 2006; Kim et al., 2013). This suggests that robots are socially perceived in a manner similar to humans (Hendriks et al., 2011; Stroessner and Benitez, 2019) and people’s needs can no longer be met by the functionality of other non-robotic tools; a robot must have social and emotional value. The introduction of perceptual design has greatly increased the public’s acceptance of robots (Kirby et al., 2010; Hwang et al., 2013).

Japanese scholars proposed the Kensei evaluation method, which is used to quantify the characteristics of a target at the perceptual level such that the characteristics can be evaluated through mathematical analysis (Matsubara and Automation, 1996). This method has also been applied in several other HRI studies (Kanda et al., 2001; Takeuchi et al., 2006; Mitsunaga et al., 2008; Usui et al., 2008; Hendriks et al., 2011; Tay et al., 2014). These scholars have collected and analyzed the perceptual vocabulary used in HRI to evaluate the interaction process or the robot. One part of the vocabulary described the feeling involved with the interaction, whereas the other part described the personality of the robot. Other scholars examining robots’ personalities have directly applied the human psychology model to characterize the personalities of robots (Kim et al., 2008) or used the personality trait model of human psychology as a reference to increase or reduce the number of items (Eysenck, 1991; Tapus et al., 2008). A more common reference is to the Big Five Theory, which is widely used to evaluate human personality traits (Norman, 1963; Barrick and Mount, 1991; Cattell and Mead, 2008). With reference to this theory, Hwang et al. (2013) performed an analysis in which 13 personality trait adjectives were extracted and used to evaluate differences in perceptions of several robots’ personalities based on the robots’ appearances. These studies reveal a key method of evaluating perceptions of robots’ personalities.

The appearance, gestures, expressions, and voices of robots in social interactions are called social cues (Saygin et al., 2012; Hwang et al., 2013; Niculescu et al., 2013; Stanton and Stevens, 2017; Menne and Schwab, 2018). They can be classified as verbal or non-verbal (Tojo et al., 2000; Mavridis, 2015). In human interaction, non-verbal communication can explain and supplement verbal communication, improve communication efficiency, and make dialog more lively (Mehrabian and Williams, 1969; Argyle, 1972). In HRI, these social cues also affect people’s perception and opinion of robots (Fong et al., 2003; Hancock et al., 2011). Robots’ appearance has been gradually developed and perfected since the uncanny valley theory was advanced (Mori et al., 2012), and most robots currently on the market have a humanoid appearance. Regarding facial expressions, mechanical and humanoid robots do not have complete facial expression functionality; expressive light is typically used as a substitute for facial expressions (Collins et al., 2015; Baraka et al., 2015, 2016; Baraka and Veloso, 2018; Song and Yamada, 2018; Westhoven et al., 2019). Our previous works have comprehensively discussed the use of voice and expressive light in social robots, providing a functionality reference for applications in three occupational fields: shopping reception, home companion, and education (Dou et al., 2020a,b, 2021).

However, the difference of gesture design in various applications were not discussed in our previous study. In non-verbal communication, gestures are pivotal because they both transmit information (Kendon, 1988; Gullberg and Holmqvist, 1999) and affect social cognition and persuasiveness (Maricchiolo et al., 2009). One study reported that in HRI, an instance of non-verbal communication is more effective than an instance of verbal communication (Chidambaram et al., 2012). A robot’s gestures also have the potential to shift cognitive framing, elicit specific emotional and behavioral responses, and enhance task performance (Ham et al., 2011; Lohse et al., 2014; Saunderson and Nejat, 2019). Currently, gestures of robots are quite basic. For example, robot Pepper often use wave, hand shake, nod, bow or arm swaying, etc. (Hsieh et al., 2017; De Carolis et al., 2021). Some robots even have no flexible and complete fingers to represent numbers (Kim et al., 2007; Chidambaram et al., 2012). In real industrial applications, the situation is even worse. Therefore, it is important to make the gestures of robots more comprehensive and meet various needs, thereby improving the interaction of different applications.

Studies on robot gestures can be divided into two categories: (1) those exploring the influence of physical parameters, such as the amplitude, frequency, speed, smoothness, and duration of a single gesture on the user’s psychology (Kim et al., 2008; Riek et al., 2010) and (2) those exploring the effects of various types of gestures (Breazeal et al., 2005; Nehaniv et al., 2005; Chidambaram et al., 2012; Huang and Mutlu, 2013). Evidence on the second category of research is more abundant. This is because robots must complete a series of behaviors in HRIs; moreover, multiple types of gestures may be involved and undergo several changes. Therefore, investigating various gesture types and their impacts is preferable to studying a single type of gesture. The mainstream view is that gestures can be divided into four categories, as proposed by McNeill (1992): (1) deictics, which indicate concrete objects or space; (2) iconics, which relate to concrete objects or events; (3) beats, which include short, quick, and frequent hand and arm movements; and (4) metaphorics, which promote visualization of abstract concepts or objects through the presentation of concrete metaphors. This theory was applied to robot research by Huang and Mutlu (2013), who observed that all types of gestures affect user perceptions of the robot’s narrative performance.

We discovered a considerable knowledge gap in relation to the design of gestures in social robots, with other scholars also noting that robot gestures shift cognitive framing, elicit specific emotional and behavioral responses, and improve task performance (Ham et al., 2011; Lohse et al., 2014; Saunderson and Nejat, 2019). Two shortcomings in these studies are notable. First, environmental differences were not considered. One study reported that even for robots of the lowest level (e.g., cleaning robots), regarding the household as an environment is necessary, and the influence of factors, such as people, products, and activities, in that environment must be considered (Forlizzi and DiSalvo, 2006). Users exhibit varying attitudes toward, preferences for, and degrees of trust in robots depending on the environment (Huang and Mutlu, 2013; Savela et al., 2018). Scholars have also begun to study users’ preferences for and trust in robots according to specific occupational fields (Huang and Mutlu, 2013; Savela et al., 2018), such as shopping reception (Aaltonen et al., 2017; Bertacchini et al., 2017), education (Robins et al., 2005; Tanaka and Kimura, 2010; Cheng et al., 2018), home-based care (Broadbent et al., 2018), and security or healthcare (Tay et al., 2014). With the introduction of robots into numerous occupational fields, the interactive environment has also changed, which suggests that future robot gesture studies should consider occupational fields to promote design customization. Second, most robot gesture studies have centered on task performance, persuasiveness, and trust in association with distinct gestures (Li et al., 2010; Lee et al., 2013; Saunderson and Nejat, 2019). The effects of individual gestures on the sociality of robots (i.e., differences in perceptions of personality traits) have not been discussed in detail.

The purpose of the study are as follows: (1) Study whether the four types gestures appear frequently in these four applications (shopping reception; home companion; education; security). (2) Discuss whether different gestures significantly affect robots’ perceived personalities. (3) Propose the design guidance of gestures for these four applications. In our previous study (Dou et al., 2021), we have confirmed that the voices and expressive lights of social robots enable the perception of distinct personality traits. Moreover, the optimal configuration of voice and lighting design parameters for social robots applied in the four fields of interest has been determined (Dou et al., 2020a,b, 2021). New discoveries on robot gestures are also presented herein.

## Materials and Methods

### Stage 1: Gesture Extraction

#### Dialog Script

**Figure 1:**
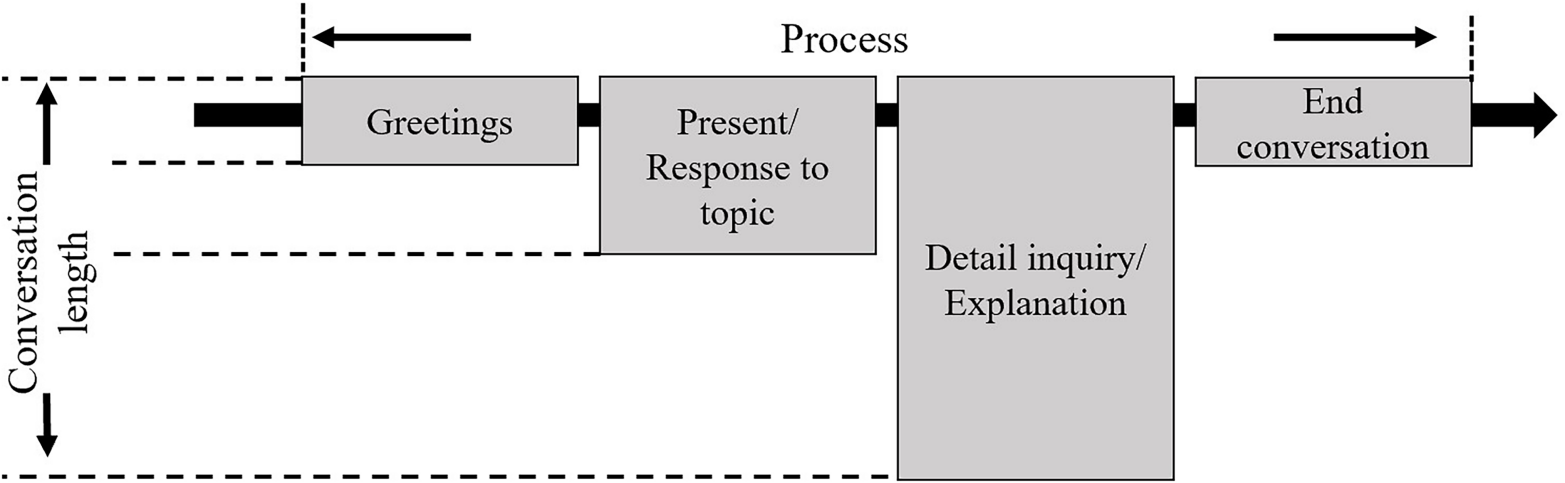
Dialog structure.

Some scholars have established scripted dialogs to plan robot behavior during HRI (Hinds et al., 2004; Louie et al., 2014; Hammer et al., 2016). In the present study, to examine the differences associated with dialog content, we defined the dialog structure and length consistently for the four applications (Figure 1).

The narration is typically the most critical part of communication (Huang and Mutlu, 2013); it is the stage with the greatest amount of information transfer and is often accompanied by non-verbal communication using various methods. Therefore, we mainly focused on recording and examining gestures displayed during this component. In accordance with the dialog structure, we devised a dialog script for each of the occupational fields (shopping reception, home companion, education, and security).

In the script of the shopping reception robot (Supplementary Table S1), the robot acts as shopping reception that is questioned by customers. The customers plan to buy a refrigerator, and the robot conveys information concerning product functions and features. In the dialog script of the home companion robot (Supplementary Table S2), the robot acts as a family caregiver communicating with an elderly person, relaying the details of a dinner recipe and the nutritional benefits of the food. In the dialog script of the educational robot (Supplementary Table S3), the robot acts as a teacher explaining the concept of the golden ratio to a user (student). In the security dialog, the robot acts as a home security manager giving a solution of house fire hazard for house owner (Supplementary Table S4).

#### Observation and Extraction

**Figure 2:**
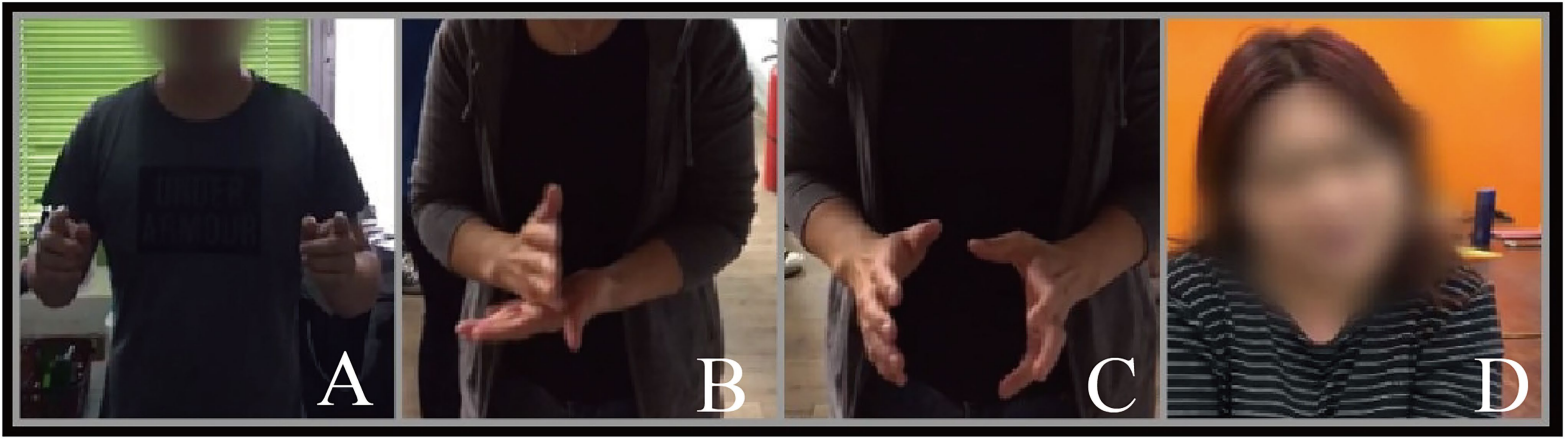
Selected screenshots from the observation (**A**: longer segment, **B**: chop into pieces, **C**: washing, **D**: titling head).

Observation is a method in which researchers use their senses and auxiliary tools to scrutinize a target for a certain research purpose, outline, or observation table (Baker, 2006). To capture gestures in real occupational environments, we developed dialog scenarios for the four occupations. We recruited 26 participants (four experts in each of the four fields and 10 members of the public; Table 1). After informed consent was obtained, video recording and observation were conducted (Figure 2). During the observation, the participants were involved in the dialog scenarios, with a staff member acting as the user. The participants were asked to express the content as clearly as possible.

**Table 1:** Background information of the participants.

Occupational field	Experts		General public
	Items	Experience	Items
Shopping reception	Four shopping receptions from Tatung 3C Shop	2*(10-year experience) 2*(5–10-year experience)	10 people aged 20–30 years old no experience
Home companion	Four nurses from National Taipei University of Nursing and Health Sciences	3*(10-year experience) 1*(5–10-year experience)	
Education	Four teachers from Department of Industrial design, Tatung University	3*(10-year experience) 1*(5–10-year experience)	
Security	Four community securities from Dongguan	1*(10-year experience) 3*(5–10-year experience)	

Each of the 16 experts only participated in one observation (in the relevant occupational field). The 10 members of the general public were required to participate in observations in all four fields. Because the experts had at least 5 years of work experience, we believe that their behavior in the interaction was professional. Therefore, they only participated in observations related to the applications in which they had experience. The experts’ gestures were assigned a high priority for consideration in the behavioral analysis. Participants from the public were not experienced practitioners in these fields; they had no experience-related impact on gestures for any of these domains. Therefore, we requested that they participate in all observations of four applications, analyzing their behavior as supplementary content.

On the basis of our expected results and the actual observations, we categorized the gestures associated with the four occupational fields, correlating them with specific elements of the dialog (Supplementary Table S5). Some gestures only appeared when specific information that was highly relevant to the overall dialog content was discussed (Huang and Mutlu, 2013). These gestures were defined as Type 1 (T1) gestures and included gestures in the deictics, iconics, and metaphorics categories. Gestures were labeled as (A) shopping reception, (B) home companion, (C) education, and (D) security. Another type of gesture, denoted as Type 2 (T2) gestures, recurred randomly and was unrelated to any specific information. Because the mode of expressing such gestures did not vary among the four fields, we labeled them (F). Regarding the extraction criteria of certain gestures, we used the number of people in the expert group greater than or equal to 50% (i.e., 2 out of 4) and the overall level greater than or equal to 50% (7 out of 14). Such gestures were considered more likely to occur in the interaction than were other gestures. Science too many items of gesture extraction cannot be listed in detail in the text, we list them in Supplementary Table S5. Supplementary Table S5 records the complete gesture extraction results, both selected and unselected.

The T1 gestures extracted for robots in four occupational fields were as follows: in the shopping reception dialog, A1 (“*this refrigerator*”) (one hand palms out, point to the refrigerator), A2 (“*three, four people*”) (put up 3 or 4 finger), A3 (“*level 1*”) (put up one finger), A4 (“*ice maker*”) (two palms facing each other in front of the chest represent a space), and A5 (“*fruit and vegetable storeroom*”) (the same as A4) all met the screening requirements. In the home companion dialog, B1 (“*three to four spareribs*”) (put up 3 or 4 fingers), B3 (“*add […] scallions and salt*”) (fingers of one hand pinching and shaking), B6 (“*one person*”) (put up a finger), and B7 (“*chopped into small pieces*”) (One hand is placed horizontally and the other is moved up and down) fulfilled our screening conditions. In the education dialog, C2 (“*a line*”) (put up a finger), C3 (“*two segments*”) (put up two fingers), C4 (“*shorter segment*”) (two index fingers facing each other in the space), C5 (“*longer segment*”) (the same as C3 but longer spacing) and C6 (“*this is called*”) (palms out and point to the screen) satisfied our screening requirements. In security dialog, D1 (“*the water heater*”) (palms out and point to the weather heater), D3 (“*2.4 meters*”) (point out 2 fingers then 4 fingers), D4 (“*an exhaust fan*”) (use the thumb and index finger of both hands to represent a circle in space), D5 (“*the window*”) (point to the window), D6 (“*there are…*”) (point to the sundries) were selected. These gestures are considered relatively common in the fields of interest. Although some individuals have expressed other information through gestures, such as A6 (“*−18 degrees*”) (two hands cross for 10 then put up one thumb and index finger for 8), A8 (“*rapid refrigeration*”) (one finger circle fast in space), A11 (“*keeps the meat fresh*”) (palms together), B8 (“*washing*”) (two hands move up and down relative to each other), and D2 (“*the height*”) (one hand placed horizontal by the side). They did not meet the screening criteria and thus could not be used as a reference for the robot gesture design in the Stage 2: experiment.

For the T2 gestures that were unrelated to the dialog content, F1 (“*beat gestures*”) (one or two hands shake intermittently) met the extraction criteria. Only one person used F3 (“*put up a finger*”) (hold a finger in the air and remain still for a long period of time) in observation. Although F2 (“*tilting head*”) (tilt the head to one side and hold it for a longer period of time) did not meet the criteria, the occurrence in the public group was close to half. The reasons underlying the screening of gestures that occurred at this stage of the study are presented in the Discussion.

### Stage 2: Robot Experimentation

We developed the robot gestures used in the experiment based on the results of the preliminary study. Experiments were designed to verify the impacts of the T1 and T2 gestures on perceptions of robots’ personality traits in the four occupational fields.

#### Participants

The study was permitted by National Taiwan University: Office of Research and Development. The participants were recruited from internet. Interested persons were invited to the school for assessment. To ensure that the participants could easily complete the experiment, the following inclusion criteria were applied: (1) Possesses a basic understanding of social robots (has watched or personally interacted with such a robot) and (2) has no physical or cognitive impairment that could prevent completion of the experiment. Subjects who meet the requirements were asked to sign an informed consent form. Only signed subjects were able to participate in the experiment. After the experiment, subjects were rewarded with NT$200. According to our assessment, 3 had high interactive experience, 24 medium, and 7 low. Of the 34 questionnaires returned, 6 were invalid and thus excluded from further analysis. A total of 28 valid responses belonging to 13 male and 15 female participants [*M* = 31.45, standard deviation (SD) = 8.85] were analyzed.

#### Experimental Design

**Figure 3:**
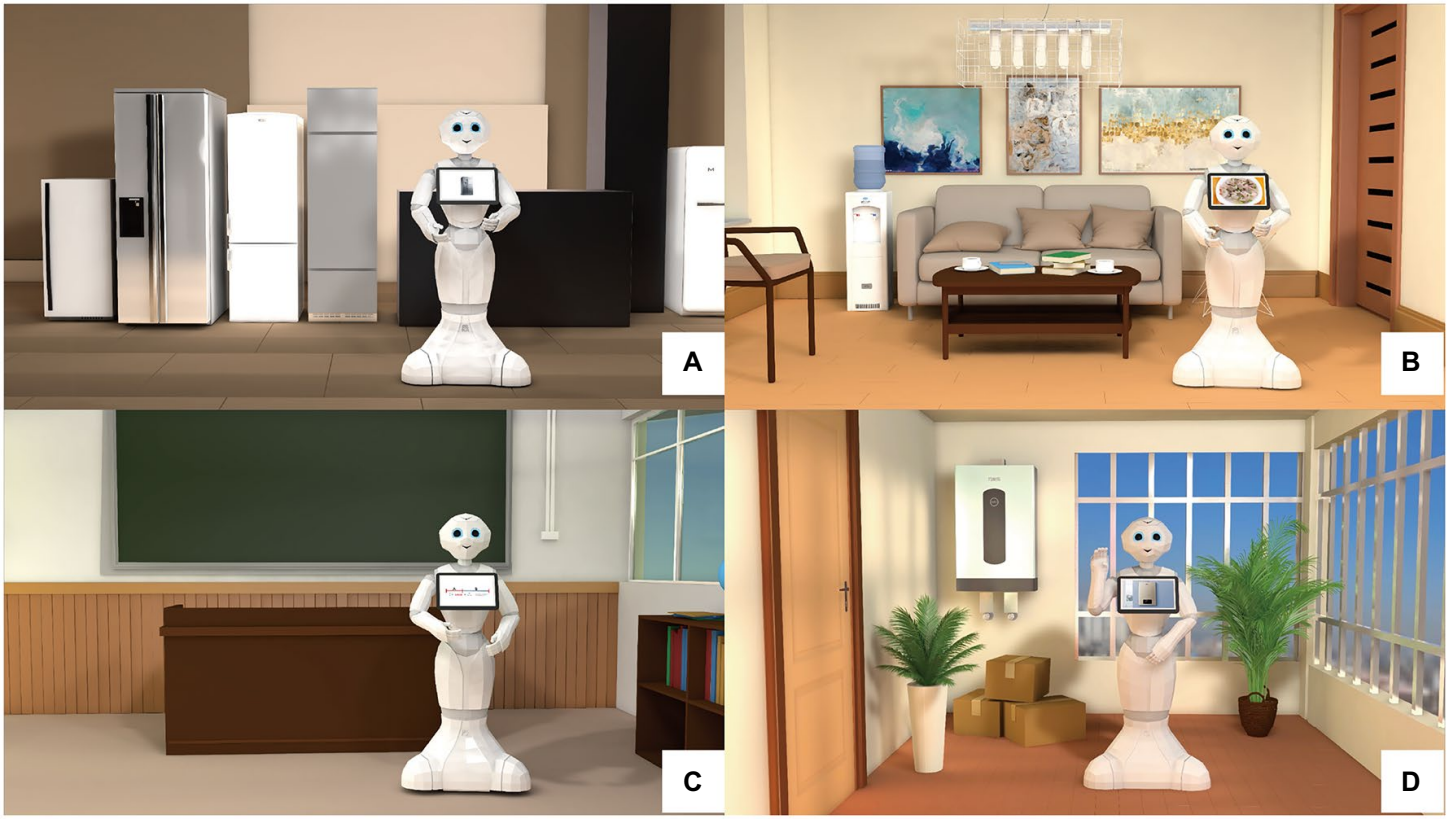
Video captures from the video HRI. **(A)** Shopping reception; **(B)** home companion; **(C)** education; **(D)** security.

Because this experiment involved the design of robot gestures, we selected Pepper, a semi-humanoid robot, as the sample model. This is because compared with other common robots (e.g., NAO and Alpha), Pepper has a complete hand structure that is relatively humanlike, enabling the accurate display of various gestures. We employed a video HRI method; that is, we produced robot-centered videos for the experiment. In recent years, this method of using real robots as models in animation has been acknowledged by scholars (Takayama et al., 2011; Walters et al., 2011; van den Brule et al., 2014). In robot-related experiments, computer-based simulations are typically easier to manipulate than are physical prototypes and exhibit greater flexibility (Figure 3; Bartneck et al., 2004).

Each video contained three key design components: the robots’ gesture, sound, and background environment. To ensure that the study remained focused on the robots’ gestures, vocal interference was eliminated, and to ensure that the vocal pitch and dialog speed were the same across occupational fields, we used synthetic speech (iFLYTEK Voice) to provide uniform voice processing. According to the dialog scenarios for the four fields, we introduced background furniture that suited the respective situations. In the gesture observation, two types of gestures were defined: T1 (deictics, iconics, and metaphorics), which occurred when topic-specific information was conveyed, and T2 (beat gestures), which randomly recurred many times. To examine the impact of these two types of gestures on perceptions of the robots’ personality traits, we established three levels of gestures as follows: level 1 (no gestures), level 2 (T1 gestures only), and level 3 (T1 + T2 gestures). The independent variable of the experiment is gesture type (level 1; level 2; level 3) and application fields (shopping reception; education; home companion; security). Given that three experimental variants corresponded to each of the four occupational scenarios, a total of 12 experimental scenarios were designed. The dependent variables of the experiment were 20 perceived personality adjectives and the overall acceptance of the robot.

#### Experimental Steps

The experiment was set up in our laboratory. In accordance with the video HRI approach, the participant interacted with the robot in the video. Participants were asked to interact with scripted tasks of different applications. And the robot’s responses were controlled as per the Wizard of Oz experimental method (Dahlbäck et al., 1993). The Wizard of Oz experiment method means that the robots in the experiment were not fully automatic and relied on the control by research staff. In HRI researches, some scholars use this method to control and simulate robots’ behavior when the robot is not autonomous and intelligent enough (Riek, 2012; Marge et al., 2017). In our experiment, whenever the user finished speaking, the experimental staff controls the robot to answer on the screen through another computer. After each animation interaction, the participants were required to complete questionnaires before interacting with the next animation. The play order of the experimental animations and the order of the questionnaire items were randomized to prevent any bias in the experimental results. Each video interaction is within 2 min, and then the subjects have 2 min to fill out the questionnaire, and there is a 10-min break in the middle of the experiment. The total experiment is about 1 h (12*4 + 10 = 58).

#### Questionnaire

In HRI researches, Godspeed questionnaires are a method for evaluation (Bartneck et al., 2009). However, some items are not evaluation of perceived personalities. For example, *(Machinelike–Humanlike)* in *anthropomorphism* and *(Mechanical–Organic)* in *Animacy* are more fits for evaluations of physical properties. Therefore, we referred to the methods of scholar (Hwang et al., 2013) and selected the adjectives of perceived personality traits. On the basis of the literature, we compiled an initial list of relevant adjectives describing perceptions of the robots’ personality traits (Norman, 1963; Barrick and Mount, 1991; Eysenck, 1991; Cattell and Mead, 2008; Kim et al., 2008; Tapus et al., 2008; Hwang et al., 2013). After expert discussion, adjectives with high similarity were merged, and finally, 20 items were generated (Table 2). For understanding the user’s overall evaluation of the robot, the *Acceptance* item was added. Which means: *I like this robot and am willing to accept its services.* All 21 items were used to evaluate 12(3*4) experimental samples. Participants were required to score all 21 items after each interactive video. The questionnaire uses a five-point Likert scale. A score of 5 indicates that the robot fits the description of the item, and a score of 1 indicates that the robot does not match.

**Table 2:** Questionnaire items.

1. Extroverted	6. Smart	11. Emotionally stable	16. Decisive
2. Confident	7. Creative	12. Adapted	17. Independent
3. Friendly	8. Nimble	13. Professional	18. Powerful
4. Happy	9. Talkative	14. Active	19. Rational
5. Helpful	10. Hardworking	15. Warm	20. Fashionable
21. Overall acceptance			

## Results

### Factor Analysis

To assess the influence of gestures on user perceptions, we conducted factor analysis to reduce the dimensionality of the data (20 adjectives). The first-order factor analysis (Kaiser–Meyer–Olkin value = 0.937, *p* < 0.000) revealed that “20 Fashionable” lower than 0.5 in all three dimensions; hence, it could not be classified and was eliminated. The remaining 19 words were subjected to second-order factor analysis (Kaiser–Meyer–Olkin value = 0.937, *p* < 0.000). These words were divided into three dimensions, as detailed in Table 3. According to the reliability analysis, the F1, F2, and F3 Cronbach’s alpha values were 0.915, 0.795, and 0.780, respectively.

**Table 3:** Factor analysis results.

Items	Factor			Cronbach’s alpha
	1	2	3	
8. Nimble	0.818			0.915
15. Warm	0.817			
9. Talkative	0.813			
14. Active	0.756			
7. Creative	0.724			
1. Extroverted	0.706			
4. Happy	0.691			
3. Friendly	0.595			
11. Emotionally stable		0.753		0.795
19. Rational		0.629		
13. Professional		0.579		
12. Adapted		0.567		
5. Helpful		0.520		
10. Hardworking		0.518		
6. Smart		0.511		
16. Decisive			0.766	0.780
17. Independent			0.754	
18. Powerful			0.639	
2. Confident			0.613	
Accumulated explanatory rate	29.38%	44.40%	59.37%	

Factor analysis revealed that “8 Nimble,” “15 Warm,” “9 Talkative,” “14 Active,” “7 Creative,” “1 Extroverted,” “4 Happy,” and “3 Friendly” were contained within F1. These words were likely to be used to describe the robot’s attitude toward the outside world (i.e., the robot’s sociality). Thus, we termed F1 the social factor. F2 included the “11 Emotionally stable,” “19 Rational,” “13 Professional,” “12 Adaptable,” “5 Helpful,” “10 Hardworking,” and “6 Smart,” items. These words tended to be used to express robots’ ability to work or perform tasks. Thus, F2 was named the competence factor. F3 comprised the items “16 Decisive,” “17 Independent,” “18 Powerful,” and “2 Confident,” which tended to be employed to describe status in an interaction; therefore, we named F3 the status factor. Our identification of status in addition to the two well-known dimensions of sociality and competence is notable. This is further explained in the Discussion.

### Multivariate Analysis of Variance

The independent variables, namely, gesture type (none, T1, or T1 + T2) and occupational field (shopping reception, home companion, education, and security), were subjected to a 3 × 4 multivariate analysis of variance. Dependent variables consisted of F1 (social), F2 (competence), F3 (status), and overall acceptance.

#### Main Effect

The multivariate analysis of variance results (Table 4) suggests that occupational field exerted a significant effect only on F3 status, *F* (*3*, *6*) = 2.98, *p* < 0.05. No significant difference was observed between shopping reception (*M* = −0.08, SD = 1.11), home companion (*M* = −0.06, SD = 0.92) and security (*M* = −0.13, SD = 0.80). The education score (*M* = 0.27, SD = 1.10) was significantly higher than other three. This indicated that in terms of the status factor (F3), shopping reception, home companion and security had relatively similar results. Thus, the sense of status the education robot conveyed in the interaction was notably greater than that of the other three robots.

**Table 4:** Results of MANOVA.

Result of MANOVA							
				Factors of dependent variable			
				Social	Competence	Status	Acceptance
Means and standard deviations	Shopping reception		N	−0.67	−0.58	−0.40	2.86
				(0.83)	(0.66)	(1.09)	(1.21)
			T1	−0.37	0.34	−0.03	3.14
				(1.09)	(0.99)	(1.08)	(1.18)
			T1 + T2	0.92	−0.05	0.19	3.89
				(0.65)	(1.33)	(1.11)	(0.96)
	Home companion		N	−0.68	−0.08	−0.42	2.75
				(0.81)	(0.90)	(1.03)	(1.18)
			T1	0.36	0.27	−0.05	3.96
				(0.73)	(1.08)	(0.86)	(0.79)
			T1 + T2	0.55	0.01	0.29	3.75
				(0.80)	(1.14)	(0.73)	(0.93)
	Education		N	−0.97	−0.19	0.18	2.18
				(0.92)	(0.95)	(1.28)	(0.82)
			T1	0.01	0.17	0.35	3.68
				(0.89)	(0.95)	1.03	(0.77)
			T1 + T2	0.59	0.12	0.29	3.79
				(0.99)	(1.35)	(0.99)	(0.87)
	Security		N	−0.70	−0.51	−0.45	2.61
				(0.67)	(0.57)	(0.68)	(0.79)
			T1	0.24	0.38	−0.16	3.79
				(0.27)	(0.64)	(0.71)	(0.57)
			T1 + T2	0.71	0.13	0.21	4.07
				(0.54)	(0.70)	(0.88)	(0.54)
*F*-values and effect size ( $\eta_{p}^{2}$ )	Main effect	Application fields (AF)	F	1.34	0.44	2.98^*^	1.95
			$\eta_{p}^{2}$	0.012	0.004	0.027	0.018
		Gesture type (GT)	F	93.95^**^	11.93^**^	7.98^**^	62.82^**^
			$\eta_{p}^{2}$	0.367	0.069	0.047	0.279
	Interactive effect	AF*GT	F	2.73^*^	0.87	0.59	3.01^*^
			$\eta_{p}^{2}$	0.048	0.016	0.011	0.053

N, no gesture; T1, robot only with T1 gestures; T1 + T2, robot with T1 and T2 gestures.

* *p* < 0.05;

** *p* < 0.001.

The gestures significantly affected F1 social, *F* (*2*, *6*) = 93.95, *p* < 0.001; F2, *F* (*2*, *6*) = 11.93, *p* < 0.001; F3, *F* (*2*, *6*) = 7.98, *p* < 0.001; and acceptance, *F* (*2*, *6*) = 62.82, *p* < 0.001. Regarding the social factor (F1), the no gestures category (*M* = −0.76, SD = 0.81) registered a significantly smaller effect than the T1 category (*M* = 0.06, SD = 0.83) and the T1 + T2 category (*M* = 0.69, SD = 0.77). T1 also had a significantly smaller effect than did T1 + T2. This suggests that regardless which type of gesture was employed, the robot’s sociality tended to increase, appearing more outgoing. As for the competence factor (F2), the no gestures category (*M* = −0.34, SD = 0.80) was associated with significantly lower scores than was the T1 category (M = 0.29, SD = 0.92). This demonstrates that the addition of T1 gestures can improve perceptions of the robot’s competence. But T1 and T1 + T2 (M = 0.05, SD = 1.15) exhibited no significant difference [M(I-J) = 0.24, SD = 0.13, *p* > 0.05] [M(I-J) reports the difference in Mean of two categories: Mean(T1) (I)-Mean(T1 + T2) (J)]. This demonstrates that the addition of T2 gestures in T1 exerts no such effect. Regarding the status factor (F3), no gestures (*M* = −0.27, SD = 1.06) exerted a significantly smaller effect than T1 (*M* = 0.03, SD = 0.94) and T1 + T2 (*M* = 0.25, SD = 0.93). However, no significant difference was observed between T1 and T1 + T2[*M(I-J)* = −0.22, SD = 0.13, *p* > 0.05]. This indicates that the introduction of T1 gestures enhances perceptions of the robot’s status but that the T2 gestures exert no such effect. The acceptance variable also exhibited the same pattern, with the no gestures category (M = 2.60, SD = 1.04) associated with significantly lower scores than the T1 category (*M* = 3.64, SD = 0.90), and T1 + T2 (*M* = 3.88, SD = 0.84). No significant difference between T1 and T1 + T2 was noted.

#### Interactive Effect

**Figure 4:**
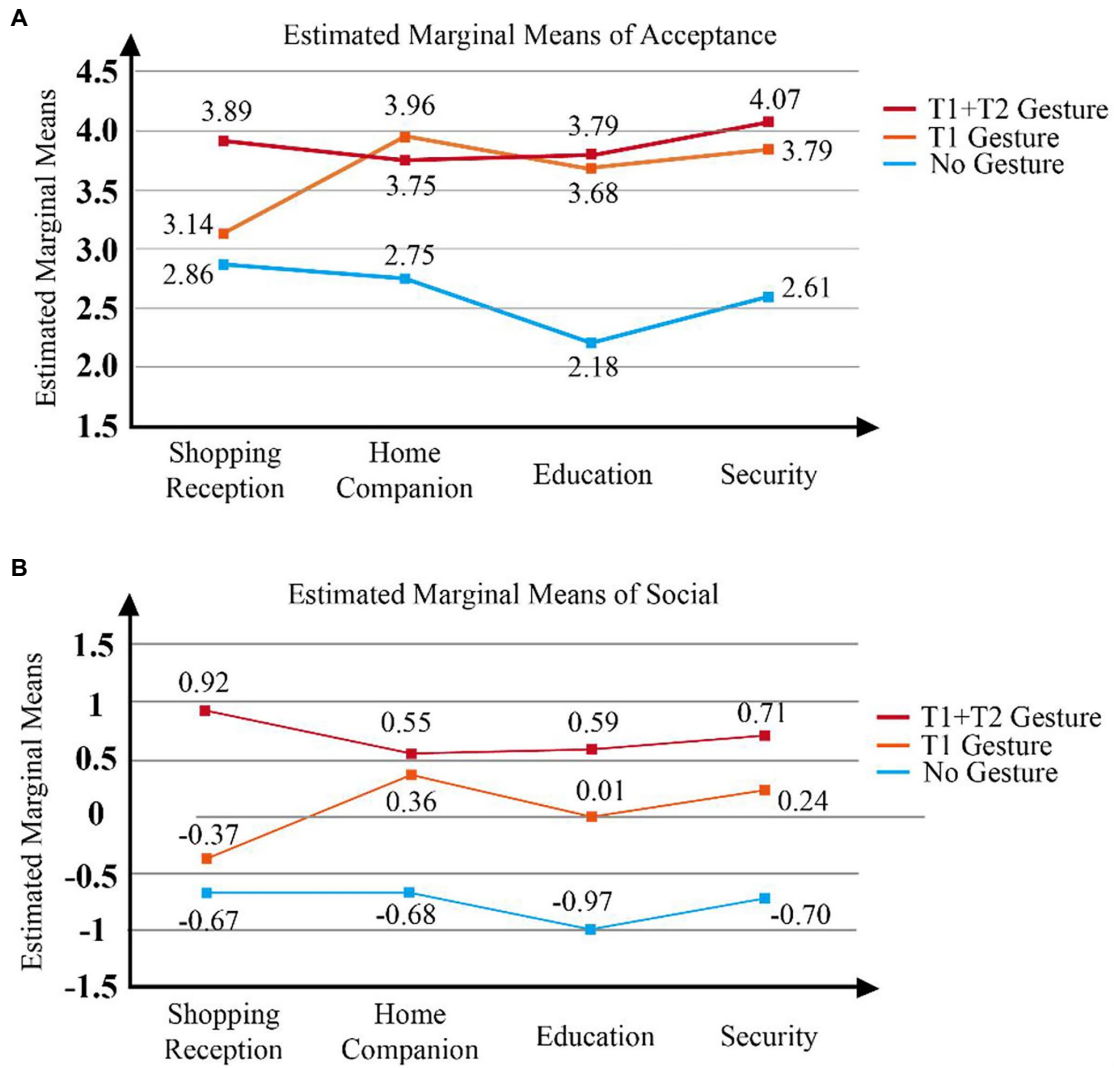
Interactive effect. **(A)** Estimated Marginal Means of Acceptance; **(B)** Estimated Marginal Means of Social.

The results indicate a significant difference among gesture types and occupational fields in terms of both F1: *F (6, 330)* = 2.73, *p* < 0.05 and Acceptance: *F (6, 330)* = 3.01, *p* < 0.05. Figure 4 shows the interactive effect of gesture types and application fields. The figures of interactive effect indicated the difference of gesture design in four application fields. Which means robot gestures should be designed differently according to applications.

The F1 results suggest no significant difference between the no gestures category (M = −0.67, SD = 0.83) and the T1 gesture category (M = −0.37, SD = 1.09) for the shopping reception robot. However, T1 + T2 substantially outperformed these two categories (M = 0.92, SD = 0.65). This implies that in the shopping reception field, the addition of T1 gestures does not improve the robot’s sociality, but the introduction of T2 gestures significantly improves the sociality factor (F1). However, in the home companion context, no significant difference between T1 (*M* = 0.36, SD = 0.73) and T1 + T2 (*M* = 0.55, SD = 0.80) was noted in terms of F1, but both significantly outperformed the no gestures category (*M* = −0.68, SD = 0.81). This suggests that the addition of T2 gestures in T1 gestures are less effective at promoting sociality in home companion applications than in shopping reception applications. Conversely, in this case, T1 gestures augmented the robot’s perceived sociality. In education contexts, the no gestures category (*M* = −0.97, SD = 0.92) registered a significantly lower F1 score than did the other two gesture categories, with the T1 category (*M* = 0.01, SD = 0.89) having a significantly lower score than the T1 + T2 category (*M* = 0.59, SD = 0.99). T1 + T2 score is also significantly higher than T1. In security fields, T1 + T2 score (*M* = 0.71, SD = 0.54) is also significantly higher than the other two, and T1 score (*M* = 0.24, SD = 0.27) is higher than no gestures category (*M* = −0.70, SD = 0.67). These indicating that with regard to education and security, T1 and T2 gestures can both significantly improve robots’ perceived sociality. This also demonstrates the differences associated with applications in distinct occupational fields.

In terms of overall acceptance, in the shopping reception application, no significant difference between the no gestures category (*M* = 2.86, SD = 1.21) and the T1 gestures category (*M* = 3.14, SD = 1.18) was noted, and T1 + T2 (*M* = 3.89, SD = 0.96) significantly outperformed these two groups because T2 gestures considerably improve sociality and therefore acceptance. However, in the home companion context, the no gestures category (*M* = 2.75, SD = 1.18) was significantly lower than both T1 (*M* = 3.96, SD = 0.79) and T1 + T2 (*M* = 3.75, SD = 0.93), and T1 and T1 + T2 did not differ significantly. In the education context, the no gestures category (*M* = 2.18, SD = 0.82) also had significantly lower scores than did the T1 category (*M* = 3.68, SD = 0.77) and T1 + T2 (*M* = 3.79, SD = 0.87), and T1 did not significantly differ from T1 + T2. In security, the performance is consistent with education and home companion, no gesture score (M = 2.61, SD = 0.79) is significantly lower than the other two, and T1 category (M = 3.79, SD = 0.57) and T1 + T2 category (M = 4.07, SD = 0.54) have no difference. In sum, although the addition of T2 gestures improves the sociality of the robot in the education and security context, the overall acceptance does not necessarily improve. This suggests that in home companion, education, and security applications, T1 gestures are more essential, whereas T2 gestures are more integral to shopping reception scenarios.

## Discussion

The purpose of this study is to explore the influence of robot gesture type on perceived personality traits in real applications and to indicate gesture design guidance in these four applications. The main findings of this study are as follows: (1) A summary about the occurrence of four types of gestures in real industry interactions. (2) Results shows that perceived personality traits of robot can be divided into three dimensions, and we explored the influence of different gesture types on these three dimensions. (3) The optimal robot gesture design guidelines for these four different applications are proposed. The specific discussions are as follows:

First, the result of observation indicated that deictics and simple iconics occurs frequently in four applications. For example, most subjects expressed deictics: “*this refrigerator,*” “*this is called…,*” and “*the window*” and simple iconics: “*level 1,*” “*3–4 spareribs,*” and “*2.4 meters*” in observation. This is because these two gestures can be performed in one hand quickly and easily. However complex numbers *“−18°C*” and “*1.618 times*” needs the cooperation of two hands. This also shows that not all iconic gestures need to appear in robot design. In addition to specific numbers, iconic gestures also mean to express specific things, such as “*ice making box,*” “*add...scallions and salt,*” “*chop into pieces,*” and “*an exhaust fan.*” This type also occurs frequently in conversation. Usually, they are used to describe specific objects or daily behaviors. The interval between gestures is very important because “*chop into pieces*” and “*washing*” are very close in context, most of the participants selected the earlier-occurring phrase “*chop into pieces*” to avoid presenting both within a short time interval. This also indicates that adequate time intervals in robots should be ensured. However, metaphoric gestures only had few occurrences, such as “*rapid refrigeration capacity,*” “*space allocation,*” “*nutritious and light,*” “*aesthetic feeling,*” and “*fire and gas poisoning.*” Most of the expected metaphoric gestures did not reach the screening threshold and therefore were beyond the scope of this study. This is because compared with iconic and deictic gestures, metaphorics are subconscious and conceptual. They cannot be expressed as intuitive and quickly as the other two. People prefer to express concrete objects rather than abstract concepts in these four applications. However, metaphoric gestures play a key role in one individual describing a foreign concept to another. In the future, we will compare professional and informal narrative content and further explore how to integrate metaphoric gestures into robot designs used in various fields. Besides, in the video, we observed that the expert and public groups frequently displayed “*beat gestures*” in all three occupational contexts, suggesting that beat gestures are common in narrative communication. However, we also noted that although “*head tilting*” did not appear as frequently as hand gestures (thus failing to meet our screening requirements), this behavior occurred often in the public group (nearly 50%). In the phenomenon of movement synchrony in human interaction, when one person unconsciously moves their head, the other tend to imitate that movement (Kendon, 1970). In the present observation, the observer likely took a head tilting to indicate that they were paying attention to the participants. The participants then subconsciously mirrored that movement. Scholars have reported that when a robot tilts its head frequently, its behavior also appears significantly more natural (Liu et al., 2012). Regarding the unconscious behavior of raising a finger, only one participant made this gesture. We believe that this type of behavior is a personal habit of that participant.

Second, perceptions of robots’ personality traits can be divided into three categories. In our previous studies, sociality and competence were considered the key categories (Dou et al., 2020a,b, 2021). However, in the present study, the additional analysis of gestures led to the discovery of a third category, status. This demonstrates that gestures and voice affect perceptions of robots’ personalities differently. The reason is that deictic and iconic gestures enhance perceptions of robots’ characteristics, including self-confidence, status, and decisiveness, thereby strengthening their perceived status. Since no metaphoric gesture were considered in experiment, the effect of this type cannot be discussed. For sociality, increases in the frequency, speed, and amplitude of any type of gesture reinforces perceptions of the robot’s extroversion (Kim et al., 2008). In the competence factor, the addition of deictic and iconic gestures improved this effect, whereas that of beat gestures produced no such impact. This is because iconics and deictics are closely integrated with words and have specific functions. The appearance of such gestures can help explain and supplement the expression of words, thus making the robot appear more competent.

Third, the best design guidelines for four applications are as follows: In the shopping reception application, the optimal design is a combination of iconic, deictic, and beat gestures. Because the application of iconic and deictic gestures alone does not exert a particularly favorable effect, the addition of beat gestures is required to significantly improve sociality and user acceptance. This is ascribable to the fact that beat gestures can significantly increase the sense of warmth, liveliness, and joy associated with a robot. These characteristics are critical for user acceptance of robots in shopping reception. Thus, for such robots, enhancing sociality through the programming of beat gestures should constitute the key design focus. In the home companion, education, and security contexts, the design recommendation is to focus on introducing iconic and deictic gestures and avoid adding an excessive number of beat gestures. This is because for these three occupational fields, the competence and status conveyed by iconic and deictic gestures are more essential. This is reflected in the fact that although the addition of beat gestures reinforces sociality in the education and security context, acceptance does not tend to improve accordingly; in other words, sociality is less important than are status and competence for educational and security robots. In home companion, beat gestures even cannot improve either robots’ sociality and acceptance. These may be because people are inclined to view these three fields as relatively quiet or serious, requiring only minimum sociality and overt enthusiasm. Instead, robots are required to be highly competent at communicating or performing tasks. In view of cost considerations, redundant gestures are a waste of funds. Hence, the industry must include and omit beat gestures from robot designs based on each gesture’s field-specific importance.

Moreover, the occupational field determines the status factor of the robot. The status required in education contexts is considerably higher than that required in shopping reception, home companion, and security applications. This perception derives from social stereotypes; sales assistants, caregivers, housekeepers, and guards are typically seen as being in supportive and passive roles, whereas teachers are usually regarded as leaders. Therefore, an image of status is required, demonstrating once more that the inclusion of iconic and deictic gestures is integral to the design of robots deployed in the education field.

The contribution of this study is to conduct research in combination with specific application fields (shopping reception, home companion, education, and security). Compared with researches only discussed both sides of human–robot interaction, the study is more in line with the actual application situation. The study on the perceived personality traits of robots also found the three dimensions of “social,” “competence,” and “status,” which is helpful for understanding and designing the perceptual value of robots in more detail. Regard of the robot gestures, the study analyzed the differences of deictic, iconic, metaphoric, and beat gestures in four applications. And finally, the guidelines for the optimized gesture design in each application were indicated, so as to facilitate more customized robot design in the future.

## Conclusion

In this study, we mainly discussed the effects of robot gestures on perceived personality traits in different applications. In the stage 1: observation, we found that metaphor gestures appear very rarely in real industrial interactions, mainly in deictic, iconic, or beat gestures. Therefore, in the second stage of the study, we explored the effect of these three types of gestures on the robot’s perceived personality traits. The result indicated that the perceived personalities of robots can be divided into three factors, among which iconic and deictic gestures significantly improve perceived competence and status. All gestures significantly improved perceived sociality. For the gesture design of robots in these four applications, adding beat gestures to robots in shopping reception can significantly improve the overall acceptance of users. However, in the other three applications, the addition of deictic and iconic gestures improved overall acceptance, while beat gesture did not. This reveals that the application fields will cause the differences in robot gesture design. The study also provides a direction for future robotics research, which is to comprehensively compare various applications, so as to propose differentiated robot design guidance.

## Data Availability Statement

The raw data supporting the conclusions of this article will be made available by the authors, without undue reservation.

## Ethics Statement

The studies involving human participants were reviewed and approved by the Research Ethics Office of National Taiwan University. The patients/participants provided their written informed consent to participate in this study.

## Author Contributions

JN worked on investigation, conducting experiment, data analysis, and article writing. C-FW guided and supervised the study and submitted article. XD assisted in investigation, conducting experiment, and data analysis. K-CL assisted in investigation and conducting experiment. All authors agree to be accountable for the content of the work and contributed to the article and approved the submitted version.

## Funding

This study was funded by Ministry of Science and Technology, Taiwan (107-2221-E-036-014-MY3).

## Conflict of Interest

The authors declare that the research was conducted in the absence of any commercial or financial relationships that could be construed as a potential conflict of interest.

## Publisher’s Note

All claims expressed in this article are solely those of the authors and do not necessarily represent those of their affiliated organizations, or those of the publisher, the editors and the reviewers. Any product that may be evaluated in this article, or claim that may be made by its manufacturer, is not guaranteed or endorsed by the publisher.
