# Supplementary material for: Designing Gestures of Robots in Specific Fields for Different Perceived Personality Traits
Source: Front Psychol. 2022 Jun 23;13:876972. doi: 10.3389/fpsyg.2022.876972 (PMC9261479; doi:10.3389/fpsyg.2022.876972)
Supplement: Supplementary file 1 [file Data_Sheet_1.docx]

Supplementary Material

**Table S1 |** Dialogue script of shopping reception.

| Application field: Shopping Reception | |
| --- | --- |
| Character: Customer (User) Reception (Robot) | |
| **Dialogue script** | |
| **Greetings** | ***Customer：***Hello |
|  | ***Reception：***Hello，may I help you？ |
| **Present/Response to topic** | ***Customer：***Do you have a refrigerator for three people？ |
|  | ***Reception：***Yes, I recommend Tatung TC-667A. |
| **Detail inquiry/**  **Narration** | ***Customer：***Well, can you give me a brief introduction. |
|  | ***Reception：***Sure. This refrigerator is our new model last year. It is specially designed for small families of 3-4 people. Its space allocation is very reasonable, with a large fruit and vegetable room and an independent ice making room. And its freezer has rapid cooling capacity. Generally, it's good to set the temperature at - 18 ℃, so it's safe to keep meat in summer. In addition, the energy efficiency level of this refrigerator is the national level-1, which is very energy-saving. |
| **End conversation** | ***Customer：***OK, I'll think about it. Thank you |
|  | ***Reception：***You’re welcome. |

**Table S2 |** Dialogue script of home companion.

| Application field: Home companion | |
| --- | --- |
| Character: Elder（User） Companion（Robot） | |
| **Dialogue script** | |
| **Greetings** | ***Elder:*** Pepper~ |
|  | ***Companion:*** Master, what do you need? |
| **Present/Response to topic** | ***Elder:*** Maybe lunch is out of control. Help me check my blood pressure. |
|  | ***Companion:*** Yes, your diastolic pressure 150, systolic pressure 95. According to the situation, radish-spareribs soup is recommended to you. |
| **Detail inquiry/**  **Narration** | ***Elder:*** OK, but I haven't cooked this dish before |
|  | ***Companion:*** The specific method is to chop them into small pieces after washing. If we calculate by one person, we need about 3-4 spareribs and a third of white radish. After cutting, put the ingredients into the pot, boil with water, and then add a small amount of scallion and salt. This soup is nutritious and light, suitable for eating too greasy or too much at noon. |
| **End conversation** | ***Elder:*** OK, If I forget the steps, I'll ask you again. |
|  | ***Companion:*** No problem. |

**Table S3 |** Dialogue script of education.

| Application field：Education | |
| --- | --- |
| Character：Student（User） Teacher（Robot） | |
| **Dialogue script** | |
| **Greetings** | ***Student:*** Excuse me. Mr. Wang. |
|  | ***Teacher:*** Well, what’s matter? |
| **Present/Response to topic** | ***Student:*** This is my final assignment. I'd like your opinion. |
|  | ***Teacher:*** OK, you should use golden ratio in this design |
| **Detail inquiry/**  **Narration** | ***Student:*** Golden ratio? |
|  | ***Teacher:*** Yes, in ancient Greece, Italy and Germany, some scholars found that when a line was divided into two segments, the longer segment was 1.618 times of the shorter segment. This is called the golden ratio. Golden ratio originated in the mathematical world, but people found that this relationship can bring aesthetic feeling, so many works of art, products and buildings adopt this relationship. |
| **End conversation** | ***Student:*** Ok, I understand. Thank you very much. |
|  | ***Teacher:*** Expect your performance. |

**Table S4 |** Dialogue script of security.

| Application field：security | |
| --- | --- |
| Character：Owner（User）home security（Robot） | |
| **Dialogue script** | |
| **Greetings** | ***Owner:*** Hello. |
|  | ***Home Security:*** Hello, I am your home security, your house needs a safety analysis. |
| **Present/Response to topic** | ***Owner:*** Ok, any questions or suggestions? |
|  | ***Home Security:*** According to preliminary scan, there is a fire hazard in the house. |
| **Detail inquiry/**  **Narration** | ***Owner:*** What exactly is it? |
|  | ***Home Security:*** Since the water heater in your home is gas-fired, the gas water heater must be installed outdoors or on a well-ventilated balcony. The height of the room is not less than 2.4 meters, and there should be an exhaust fan installed on the window. This balcony has no exhaust fan, and there are too many sundries, including paper towels, cardboard boxes, and other flammable items. This situation is likely to cause dangerous situations such as fire and gas poisoning. Therefor space renovation is recommended. |
| **End conversation** | ***Owner:*** Thank you for reminding, I will do it immediately. |
|  | ***Home Security:*** My duty! |

**Table S5 |** Numbers of gestures.

| **Num** | **Appear position in text** | **Select** | **Num** | **Appear position in text** | **Select** |
| --- | --- | --- | --- | --- | --- |
| *Shopping Reception* | | | *Home companion* | | |
| A1 | This refrigerator | Y | B1 | recommend to you | N |
| A2 | 3，4 people | Y | B2 | 3-4 spareribs | Y |
| A3 | level-1 | Y | B3 | add ... scallion and salt | Y |
| A4 | ice making box | Y | B4 | put the ... into | N |
| A5 | fruit and vegetable room | Y | B5 | nutritious and light | N |
| A6 | - 18 ℃ | N | B6 | one person | Y |
| A7 | new model | N | B7 | chop ... into small pieces | Y |
| A8 | rapid cooling capacity | N | B8 | washing | N |
| A9 | space allocation | N | B9 | the pot | N |
| A10 | energy-saving | N | B10 | suitable for | N |
| A11 | keep meat … | N | B11 | a third of white | N |
|  |  |  | B12 | boil with water, | N |
| *Education* | | | *Security* | | |
| C1 | ancient Greece, Italy and Germany | N | D1 | the water heater | Y |
| C2 | a line | Y | D2 | the height | N |
| C3 | two segments | Y | D3 | 2.4 meters | Y |
| C4 | shorter segment | Y | D4 | an exhaust fan | Y |
| C5 | longer segment | Y | D5 | the window | Y |
| C6 | This is called … | Y | D6 | this balcony | N |
| C7 | 1.618 times | N | D7 | there are… | Y |
| C8 | golden ratio. | N | D8 | paper towels, cardboard boxes, and other flammable items | N |
| C9 | originated in the mathematical world | N | D9 | is likely to cause dangerous | N |
| C10 | aesthetic feeling | N | D10 | fire and gas poisoning | N |
| C11 | this relationship | N | D11 | space renovation | N |
| *All applications* | | |  |  |  |
| F1 | bit gesture（randomly occur） | Y |  |  |  |
| F2 | head tilting（randomly occur） | N |  |  |  |
| F3 | hold up a finger（randomly occur） | N |  |  |  |
